# The value of CT-based radiomics nomogram in differential diagnosis of different histological types of gastric cancer

**DOI:** 10.1007/s13246-022-01170-y

**Published:** 2022-09-05

**Authors:** Hao Huang, Fangyi Xu, Qingqing Chen, Hongjie Hu, Fangyu Qi, Jiaojiao Zhao

**Affiliations:** 1 Department of Radiology, Sir Run Run Shaw Hospital, Zhejiang University School of Medicine, No. 3, Qingchun East Road, Hangzhou, Zhejiang China; 2 Department of Radiology, Nanxun District People’s Hospital, No.99, Fengshun Road, Huzhou, Zhejiang China; 3 Department of Radiology, Yuyao Traditional Chinese Medicine Hospital, No. 1500, Zhongshan South Road, Ningbo, Zhejiang China

**Keywords:** Gastric cancer, Histological type, Radiomics, Computed tomography

## Abstract

To establish and verify a nomogram based on computed tomography (CT) radiomics analysis to predict the histological types of gastric cancer preoperatively for patients with surgical indications. A sum of 171 patients with gastric cancer were included into this retrospective study. The least absolute shrinkage and selection operator (LASSO) was used for feature selection while the multivariate Logistic regression method was used for radiomics model and nomogram building. The area under curve (AUC) was used for performance evaluation in this study. The radiomics model got AUCs of 0.755 (95% CI 0.650–0.859), 0.71 (95% CI 0.543–0.875) and 0.712 (95% CI 0.500–0.923) for histological prediction in the training, the internal and external verification cohorts. The radiomics nomogram based on radiomics features and Carbohydrate antigen 125 (CA125) showed good discriminant performance in the training cohort (AUC: 0.777; 95% CI 0.679–0.875), the internal (AUC: 0.726; 95% CI 0.5591–0.8933) and external verification cohort (AUC: 0.720; 95% CI 0.5036–0.9358). The calibration curve of the radiomics nomogram also showed good results. The decision curve analysis (DCA) shows that the radiomics nomogram is clinically practical. The radiomics nomogram established and verified in this study showed good performance for the preoperative histological prediction of gastric cancer, which might contribute to the formulation of a better clinical treatment plan.

**Supplementary Information:**

The online version contains supplementary material available at 10.1007/s13246-022-01170-y.

## Introduction

Gastric cancer is a common malignant tumor with poor prognosis, and ranks fifth and second in incidence and mortality all over the world, which poses a serious threat to human health all over the world [1]. More than 70% of new cases of gastric cancer occur in developing countries, and in China, gastric cancer ranks second only to lung cancer in terms of the incidence of all tumors [2]. Recently, great progress has been achieved in the diagnosis process and multidisciplinary treatment strategies of patients with resectable gastric cancer, but the survival rate of those patients is still not satisfactory because of the high recurrence rate [3, 4]. Surgical resection is considered to be the main treatment for locally advanced gastric cancer, but there are still more active treatment options, such as preoperative and postoperative chemotherapy and radiotherapy. Some studies have shown that preoperative treatment of gastric cancer (neoadjuvant chemotherapy/radiotherapy + surgery + postoperative adjuvant chemotherapy/radiotherapy) has been proved to be superior to surgery alone [5, 6]. Neoadjuvant chemotherapy also led to significant increase in tumor remission rate and surgical resection rate [7, 8]. Lymph node metastasis plays a key role in selecting suitable patients for neoadjuvant chemotherapy, but the low accuracy of preoperative diagnosis complicates the selection candidate for neoadjuvant chemotherapy [9–12].

According to the Japanese classification of gastric cancer, gastric adenocarcinoma is further classified into differentiated type (including papillary adenocarcinoma and well-differentiated and moderately differentiated tubular adenocarcinoma) and undifferentiated type (including poorly differentiated adenocarcinoma, mucinous adenocarcinoma and signet ring cell carcinoma),due to significantly different clinicopathological features and prognostic differences [13]. In general, patients with undifferentiated gastric cancer have a higher risk of lymph node metastasis and a poorer survival rate than patients with differentiated gastric cancer [14]. Some studies have also shown that the diagnostic accuracy of lymph node metastasis is related to the histological type and CT stage of gastric cancer [15]. Therefore, preoperative histological diagnosis of gastric cancer is helpful for the judgement of lymph node metastasis, which might be useful to clinical treatment plan making.

At present, the histological type of gastric cancer is mainly determined by postoperative pathology. Although gastroscopic biopsy can provide certain histological information for tumor classification, it only represents local tumor tissue and will bring more damage to patients. CT is the most commonly used imaging modality for the preoperative assessment of Lymph node status, but the reported accuracy is only about 60% [16, 17], and it is difficult to distinguish different histological types of gastric cancer on CT images. Therefore, it is difficult to obtain accurate information of histological type of gastric cancer preoperatively.

Different from traditional imaging modalities, radiomics has the ability to reveal the potential features for image analysis [18]. Radiomics can quantify medical images into high-dimensional, minable data through specific extraction procedures, and support clinical decision-making through subsequent data analysis [18, 19]. Radiomics has been widely used in tumor detection, tumor subtype classification, prognosis prediction and curative effect evaluation and other fields [18–20]. For gastric cancer, radiomics has been used to predict metastasis, predict early recurrence and evaluate efficacy. However, there are few studies on the combination of radiomics features and traditional clinical features to predict the histological types of gastric cancer.

The purpose of this retrospective study was to establish and verify a radiomics nomogram based on CT to predict the histological types of gastric cancer preoperatively for patients with indications for resection of gastric cancer, which might help to identify high-risk patients for active treatment.

## Materials and methods

### Patients

A total of 171 patients with gastric cancer treated resection were collected from two independent institutions. The inclusion criteria were as follows: (1) Patients who underwent gastrectomy and were pathologically proved to be gastric cancer. (2) Gastric lesions were found by abdominal contrast-enhanced CT before operation. (3) CT examination showed complete data in DICOM format. The exclusion criteria are as follows: (1) The image quality can not meet the research, such as motion artifacts, poor gastric filling and other factors that affect the diagnosis. (2) The result of pathological diagnosis is not clear. (3) There is a history of malignant tumors outside the stomach.

A sum of 143 patients recruited from Institution I (Sir Run Run Shaw Hospital, Zhejiang University School of Medicine from January 2019 to December 2020) were randomly divided into training (n = 99) and internal validation (n = 44) cohorts. And 28 patients from institution II (Nanxun District People’s Hospital from January 2016 and October 2019) formed the external validation cohort.

### CT image acquisition

CT data acquisition and imaging parameters were listed in Table S1. All CT images were obtained with Siemens SOMATOM Definition Flash, GE Light Speed VCT and Siemens syngo CT, ranging from diaphragm to the level of iliac spine. CT enhanced scan used high pressure syringe to inject nonionic contrast medium into vein at a speed of 3–4 ml/s. Arterial phase delayed scanning was 15 s, portal venous phase delayed scanning was 70 s, and balance phase delayed scanning was 180 s.

### Traditional imaging features and clinical data

Traditional imaging features collected the average CT value of the largest axial plane of the lesion in plain scan, arterial phase, portal phase and balanced phase, and the CT difference between post-enhanced scan (arterial phase, portal phase, balanced phase) and plain scan. Those traditional quantitative imaging features were measured by two radiologists (A and B) with 15 and 10 years of working experience, respectively. Radiologists only know clinical information, not pathological results. The two doctors described the location of each tumor on their respective workstations and measured the average CT value at the largest axial plane of tumor. Any differences will be settled through concensus. Clinical data including age, sex, tumor location, carbohydrate antigen 199 (CA199), carbohydrate antigen 125 (CA125), alpha-fetoprotein (AFP) and carcinoembryonic antigen (CEA) level were collected from digital medical record.

### Image segmentation and feature extraction

The steps of image segmentation and feature extraction are completed in 3D-slice software (version 3.6.0). Since the enhancement of most gastric cancer lesions is obviously different from that of adjacent normal tissues in the portal vein phase, CT images in the portal vein phase were segmented manually in this study. Lesions were detected and located by thickening and enhancement of gastric wall. Manually segment the entire lesion and integrate the 3D volume of interest (VOI). During segmentation, necrotic areas, enlarged lymph nodes and perigastric adipose tissue were removed from VOI through multi-planar reformation (MPR) observation. MPR is to obtain two-dimensional images of coronal plane, sagittal plane and oblique plane from the original transverse axis images after reconstruction The reconstruction thickness used in this study is 5–7 mm. Image preprocess was completed with interpolation resampling algorithm at a voxel spacing of 1*1*1 to reduce the impact of the heterogeneity of CT scanners and protocols on radiomics analysis. Feature extraction was performed using 3D-slice software (version 3.6.0). Finally, a total of 850 features, including texture features, density features, shape features and filter features, were extracted from CT images.

### Feature consistency check and data standardization

The VOI of all patients was first manually segmented by a doctor with 5 years of radiological diagnosis experience. One month later,forty patients were randomly selected from 171 patients for second segmentation by the same doctors. Intra-group correlation coefficient (ICC) was used to evaluate the stability of radiomics features extracted from the two VOIs of those 40 cases. Features with ICC > 0.85 were stable enough for further analysis.

Z-SCORE standardization was applied to eliminate the influence of each characteristic numerical dimension and order of magnitude, so that it falls into a small specific interval.

### Feature selection and radiomics model building

Based on the training cohort, the least absolute shrinkage and LASSO analysis method was used to select features associated with histological types of gastric cancer. The regularization parameter λ was defined by use of tenfold cross-validation. LASSO analysis was done utilizing “glmnet” package of R software. Then, according to the selected radiomics features, a radiomics model was developed for histological prediction of gastric cancer using multivariate Logistic regression method in the training cohort and was validated in the internal and external verification cohorts.

### Construction of radiomics nomogram

A combination model was established by combining radiomics features with clinical risk prediction factors by multivariate Logistic regression method, and verified in the internal and external verification cohorts. In order to improve the value of the combination model in clinical application, this study visualized the model as an radiomics nomogram in the training cohort.

### Statistical analysis

In this study, IBM SPSS Statistics (version 20.0) was used to analyze clinical and traditional imaging feature data. Univariate analysis was used to evaluate the relationship between clinical, traditional imaging features and histological types, independent T-test or Mann–Whitney U test was used to evaluate continuous variables, and chi-square test was used to evaluate category variables. *P* < 0.05 was considered to be statistically significant.

The receiver operating characteristic (ROC) curve with the area under of curve (AUC) value were used for performance evaluation of radiomics model and radiomics nomogram. Calibration curves was used to assess the calibration of the radiomics nomogram. Decision curve analys (DCA) was used to evaluate the clinical practicability of radiomics nomograt.

## Result

### Clinical features

Table 1 showed the distribution of clinical and traditional imaging features of all patients in the training, the internal and external verification cohorts. In the cohort of training, there was no significant difference in age, sex, tumor location, CA199, AFP, CEA, CT value and enhancement amplitude of each phase between patients with differentiated gastric cancer and patients with undifferentiated gastric cancer. The value of CA125 for undifferentiated subtype was significantly higher than that of differentiated subtype in the training (*P* = 0.03), the internal (*P* = 0.008) and external (*P* = 0.026) validation cohorts.

**Table 1:** Clinical and traditional imaging characteristics of patients in the training, the internal and external validation cohort

	Training cohort (n = 99)				Internal verification cohort (n = 44)				External verification cohort (n = 28)			
	Overall (n = 99)	Differentiated type (n = 32)	Undifferentiated type (n = 67)	P-value	Overall (n = 44)	Differentiated type (n = 14)	Undifferentiated type (n = 30)	P-value	Overall (n = 28)	Differentiated type (n = 15)	Undifferentiated type (n = 13)	P-value
Age, mean ± SD, years	63.1 ± 11.3	67.3 ± 8.9	62.3 ± 12.3	0.8	64.0 ± 10.6	65.3 ± 7.2	63.4 ± 11.9	0.58	67.3 ± 10.1	67.3 ± 7.8	67.4 ± 12.7	0.98
Sex												
Female	71	27	44	0.53	30	8	20	0.48	21	11	10	0.83
Male	28	5	23		14	6	10		7	4	3	
Tumor location												
Gastric antrum	61	21	40	0.22	26	7	19	0.37	20	11	9	0.51
Gastric fundus	22	9	13		10	5	5		4	2	2	
Gastric body	16	2	14		8	2	6		4	2	2	
CT value												
Plain scan	37 (9)	37 (9.8)	37 (10)	0.89	34.5 (9.5)	32 (9.5)	35.5 (12.5)	0.29	37 (13)	37 (15)	33 (12)	0.9
Arterial phase	61 (24)	62 (19.3)	61 (27)	0.63	62.5 (20.8)	62 (22.5)	62.5 (21)	0.97	56 (19)	59 (18)	57 (14)	0.07
Portal phase	74.3 ± 19.8	72.3 ± 20.1	75.1 ± 15.3	0.54	71.9 ± 15.8	73.6 ± 16.9	71.2 ± 15.5	0.7	72.5 ± 17.6	68.5 ± 15.8	76.8 ± 18.8	0.54
Equilibrium phase	76.3 ± 20.9	71.4 ± 15.3	77.8 ± 23.2	0.16	72.5 ± 16.1	71.5 ± 15.1	73.0 ± 16.7	0.77	74.5 ± 17.6	71 ± 19.2	78.4 ± 15.3	0.76
Enhancement amplitude												
Arterial phase	27 (21)	28.5 (23.3)	26 (20)	0.54	24.5 (21.5)	28.5 (21.5)	23.5 (27)	0.57	23 (22.5)	20 (28.5)	23 (19)	0.04
Portal phase	38.7 ± 17.6	35.5 ± 20.8	38.0 ± 19.9	0.57	37.8 ± 14.9	41.2 ± 16.7	36.1 ± 13.9	0.29	29.8 ± 18.4	29.1 ± 17.8	30.6 ± 19.4	0.8
Equilibrium phase	36 (24)	36 (20.1)	36 (26)	0.37	40.7 (19.8)	37.3 (19)	41 (23.1)	0.92	38 (22.8)	35 (20.5)	40 (24)	0.03
CA199	10.5 (11.7)	11.8 (30.4)	10.1 (10.8)	0.16	11.8 (14.9)	12.3 (14.8)	11.7 (12.2)	0.18	16.3 (30)	13.7 (20)	25.3 (63)	0.79
CA125	9.4 (6.6)	9.1 (4.3)	9.8 (10.5)	0.03	12.1 (8.6)	9.4 (4.7)	16 (9.5)	0.008	9.8 (8)	6.6 (6)	12.8 (7)	0.026
AFP	2.4 (1.6)	2.2 (1.8)	2.4 (1.5)	0.94	2.6 (1.5)	2.8 (1.7)	2.6 (1.5)	0.49	3 (2)	3 (2)	3.2 (2)	0.69
CEA	2.7 (3.5)	3.2 (3.8)	2.6 (2.6)	0.16	2.4 (3.1)	3.6 (5.3)	2.1 (1.7)	0.7	2.7 (3)	3.8 (11)	3.5 (9)	0.23

The differences were assessed by independent T-test or Mann–Whitney U test or chi-square test

The underline suggests that CA125 was statistically significant in the training, the internal and external validation cohorts

### Radiomics feature selection and radiomics model development

**Fig. 1:**
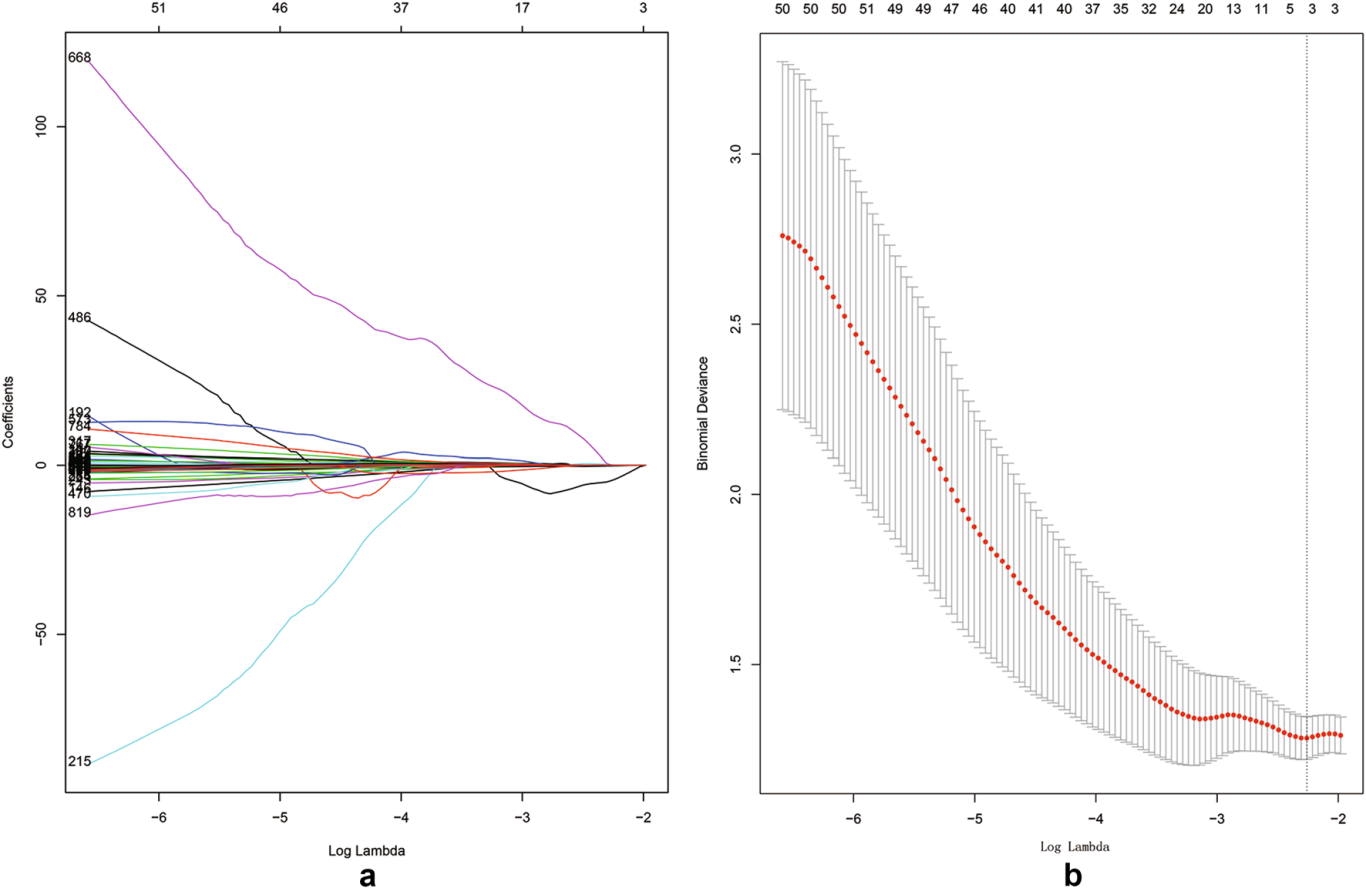
The selection process of LASSO method. **a** The best feature combination was selected by LASSO method. Each color line represents the corresponding coefficient of each feature and LASSO adjusts the parameter (Lamdba), to achieve the purpose of dimensionality reduction. **b** Selection of tuning parameter (Lamdba) in the LASSO model via tenfold cross-validation based on minimum criteria. The AUC curve was plotted against log Lamdba). Dotted vertical lines were drawn at the optimal values by using the minimum criteria and the 1 standard error of the minimum criteria (the 1- standard error criteria)

**Fig. 2:**
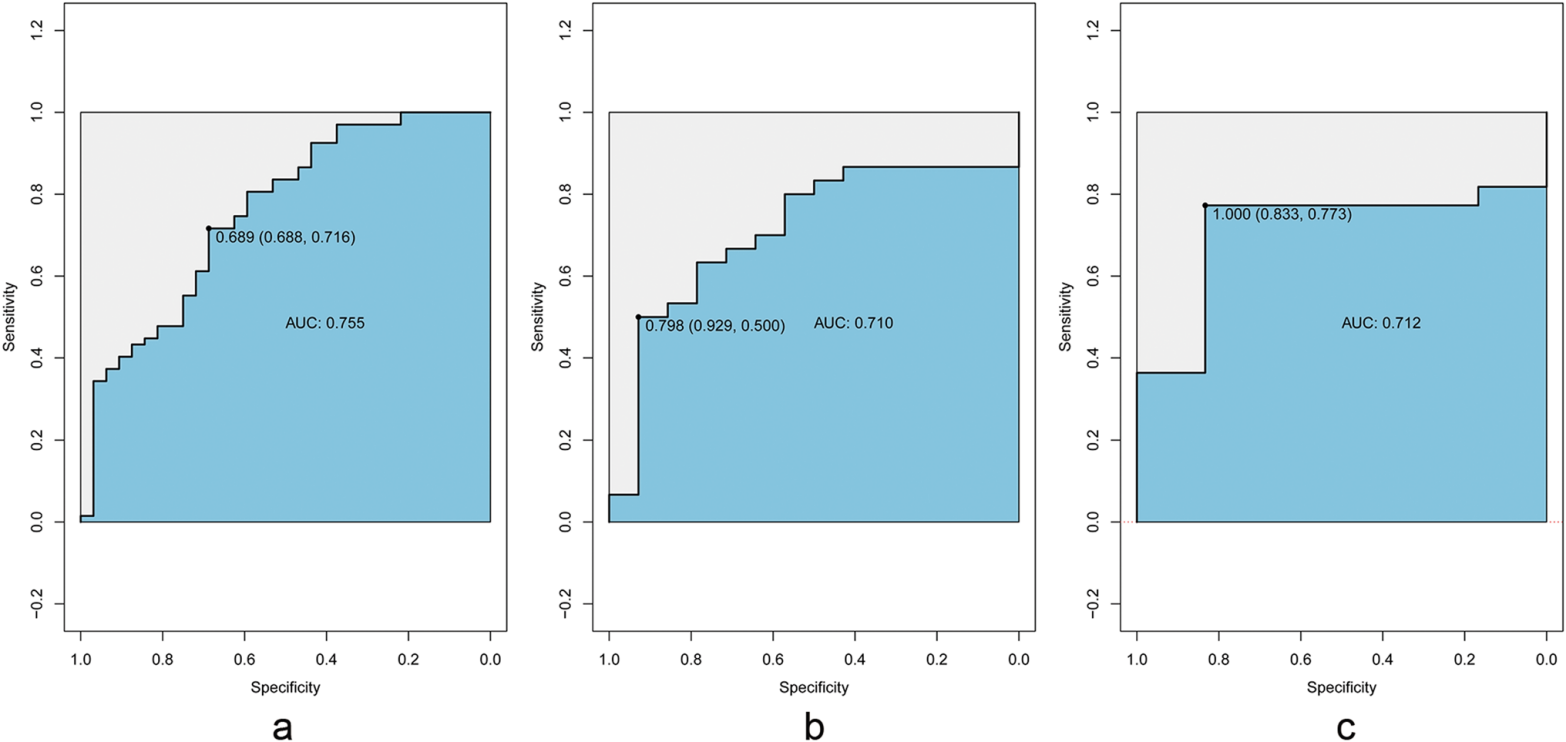
ROC curves of the radiomics model in the training, the internal and external verification cohorts. ROC curves of radiomics model in the training (AUC: 0.755, 95% CI 0.650–0.859) (**a**), the internal (AUC: 0.71, 95% CI 0.543–0.875) (**b**) and external (AUC: 0.712, 95% CI 0.500–0.923) (**c**) validation cohorts

Firstly, 824 of 850 radiomics features with good stability (ICC > 0.85) were selected for further analysis. Z-SCORE standardization was applied to eliminate the influence of each characteristic numerical dimension and order of magnitude, so that it falls into a small specific interval. In this study, the best feature combination was selected by LASSO method. The selection process of LASSO method is shown in Fig. 1. Subsequently, four radiomics features were finally selected (detailed in Table 2) to construct the Radiomics Model of patients with gastric cancer. The radiomics model has a certain prediction effect on differentiated and undifferentiated types subtypes in the training (AUC: 0.755, 95% CI 0.650–0.859), the internal(AUC: 0.71, 95% CI 0.543–0.875) and external verification (AUC: 0.712, 95% CI 0.500–0.923) cohorts (Fig. 2).

**Table 2:** Details of the four radiomics features

Feature type	Feature name	Differentiated type	Undifferentiated type
Texture feature	Glszm-small area low gray level emphasis	− 212.5 (301.3)	− 297 (274.5)
	Gldm-dependence entropy	0.092 (0.02)	0.056 (0.01)
	Gldm-dependence variance	4.82 (0.17)	4.82 (0.19)
Filter feature	Firstorder-minimum	9.22 (1.66)	8.77 (2.03)

### Predictive performance of radiomics nomogram

**Fig. 3:**
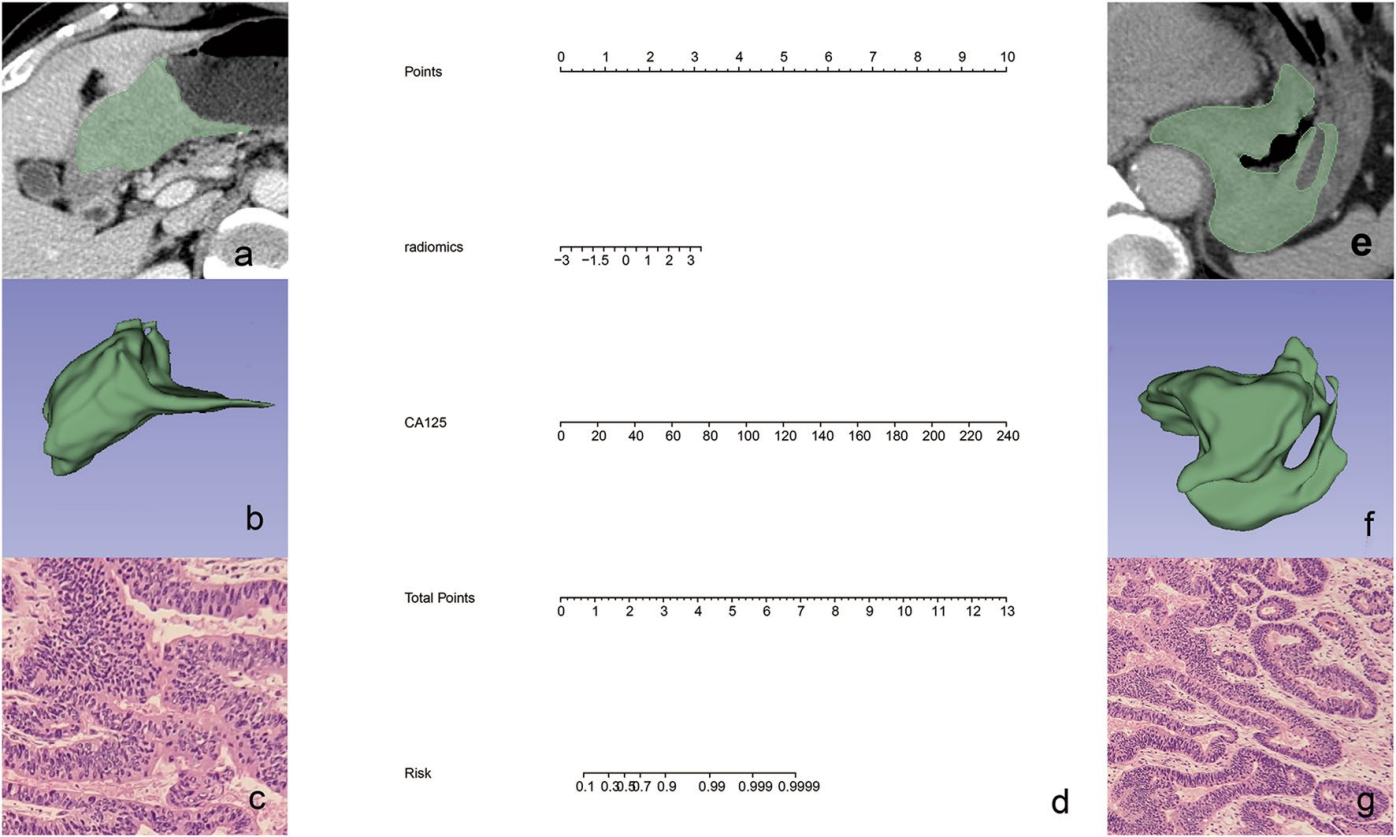
Two clinical examples of the application of radiomics nomogram. **a**, **b** A thickened lesion was observed in the gastric antrum. The radiomics score of the patient is 0.5, which means the points of nomogram is 1.2. The value of CA125 was 4.9, which means the points of nomogram was 0.25, Total point of nomogram (**d**) was 1.45, which means the probability of undifferentiated type was less than 30%. Microscopic pathological image of the surgical specimen (**c**) proved the differentiated type. **e**, **f** A thickened lesion was observed in the gastric fundus. The radiomics score of the patient is 2.1, which means the points of nomogram is 2.5. The value of CA125 was 9.0, which means the points of nomogram was 0.5, Total point of nomogram (**d**) was 3.0, which means the probability of undifferentiated type was about 90%. Microscopic pathological image of the surgical specimen (**g**) proved the undifferentiated type

**Fig. 4:**
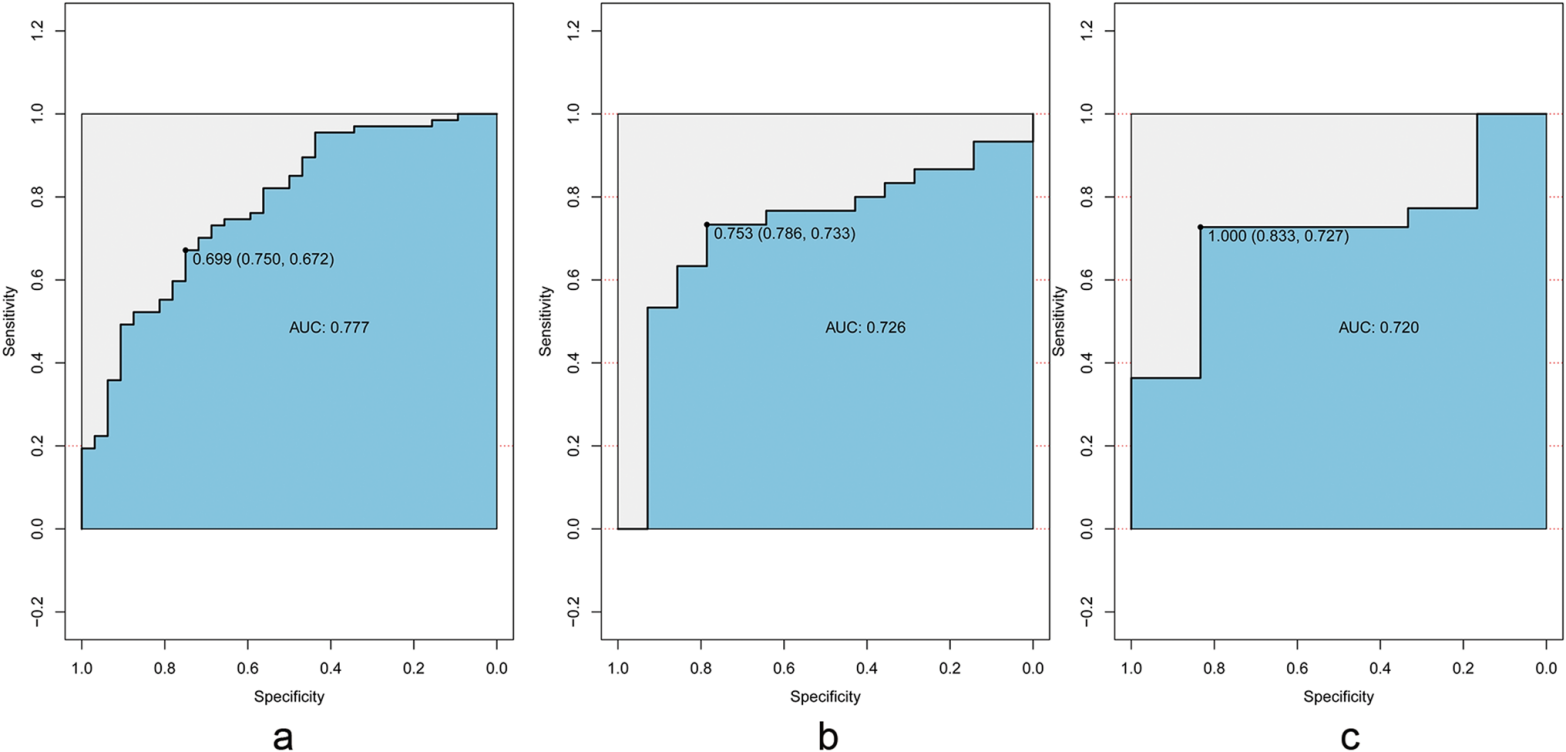
ROC curves of the radiomics nomogram in the training, the internal and external verification cohorts. ROC curves of radiomics nomogram in the training (AUC: 0.777, 95% CI 0.679–0.875) (**a**), the internal (AUC: 0.726, 95% CI 0.5591–0.8933) (**b**) and external (AUC: 0.720; 95% CI 0.5036–0.9358) (**c**) validation cohorts

**Fig. 5:**
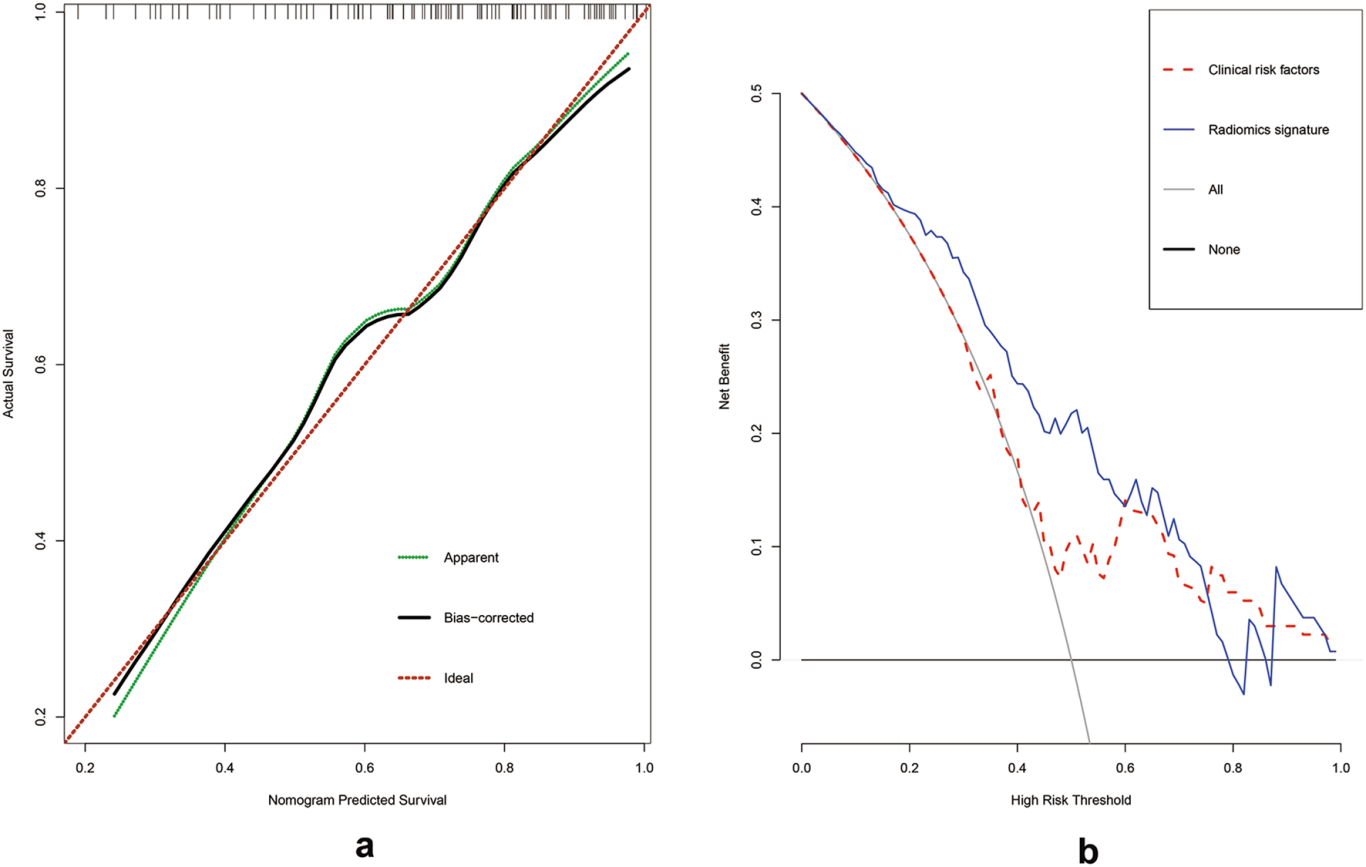
Calibration curves and Decision curve analysis of radiomics nomogram. **a** Calibration curves of the radiomics nomogram being used in the training cohort. The y-axis represented the actual undifferentiated type rate, and the x-axis represented the predicted undifferentiated type possibility. **b** Decision curve analysis of radiomic nomogram. The y-axis shows the net benefit, and the x-axis shows the risk threshold

Multivariate logistic regression analysis was conducted to build a CT-based radiomics nomogram combined radiomics features and CA125, as shown in Fig. 3. The radiomics nomogram showed good discriminant performance in predicting the histological classification of gastric cancer in the training (AUC: 0.777; 95% CI 0.679–0.875), the internal (AUC: 0.726; 95% CI 0.5591–0.8933) and external verification cohort (AUC: 0.720; 95% CI 0.5036–0.9358), as detailed in Fig. 4. The calibration curve of the radiomics nomogram shows that there is a good consistency between the actual results and the predicted results in the training cohort, as detailed in Fig. 5a. As shown in the DCA (Fig. 5b) of the nomogram, when the threshold probability is 20%-80%, it shows a greater net benefit than the treatment of all patients or no treatment.

## Discussion

Preoperative histological subtype diagnosis of gastric cancer is instructive for clinical treatment. In this study, a novel CT-based radiomics nomogram that combined selected radiomics features and CA125 was established and tested to predict the histological types of gastric cancer before operation.

The AUC of CT-based radiology nomogram in the training, the internal and external validation cohorts are 0.77, 0.726 and 0.72 respectively, which proved its potential value in preoperative histological discrimination of gastric cancer. In addition, compared with common binary judgement of predictive model, nomogram to some extent visualized the role of each feature for model judgement and provided a quantitative probability for each patients, which might improve the credibility of the prediction performance of radiomics models in clinical practice.

A total of 850 features, including texture features, density features, shape features and filter features, were extracted from CT images. The 13 shape features describe the shape and size of the tumor.The 18 density features describe the distribution of voxel-based CT intensity in tumors. The 75 texture features describe the relative positions of various grayscale on the image. Through filtering transformation, the features re-collected from density features and texture features are filter features.

Four radiomics features are selected to construct radiomics nomogram, and three of them focus on the texture features of the image: gray-level size zone matrix (GLSZM) and gray-level dependence matrix (GLDM).

First of all, Both GLSZM and GLDM are used to evaluate the similarity of image grayscale in plane or row direction. Therefore, GLSZM and GLDM reflect the local grayscale correlation, the higher the value, the greater the correlation. In this study, the greater the value of GLSZM and GLDM, the greater the grayscale correlation of the tumor image.

Secondly, the median of the three texture features of differentiated gastric cancer was higher than that of undifferentiated gastric cancer, indicating that the grayscale correlation of CT images of patients with differentiated gastric cancer was greater.

Finally, I think this may have something to do with the gap between cancer cells. Some studies have mentioned that differentiated types include papillary and tubular adenocarcinomas. Undifferentiated types include poorly differentiated adenocarcinoma, signet ring cell carcinoma and mucinous adenocarcinoma. In poorly differentiated adenocarcinomas, solid or medullary types are characterized by tight accumulation of tumor cells [14]. Therefore, in the CT images of patients with undifferentiated gastric cancer, the close accumulation of cancer cells in different forms leads to a lower grayscale correlation, which makes the values of GLSZM and GLDM smaller.

There were indeed some limitations in this study. First of all, the sample size was relatively small. Expanded sample size of prospective and multicenter external verification was necessary to further verify the performance of the nomogram in this study. Secondly, the radiomics features used in this study are only extracted from CT images in the portal vein phase, which might excluded some potential value of radiomics features. Therefore, other stages would be further studied. Finally, this study only discussed the relationship between radiomics model and histological type. In future studies, we will increase the correlation between radiomics models and local recurrence, survival and lymph node metastasis, so as to make the study more clinical practical.

## Conclusion

In this study, we accomplished CT based radiomics analysis for predicting the histological types of gastric cancer and the radiomics nomogram established in this study could roughly predict the histological type of gastric cancer and contribute to the clinical formulation of a better treatment plan.

## Supplementary Information

Below is the link to the electronic supplementary material.

Supplementary file1 (XLSX 13 KB)

## Abbreviations

CT: Computed tomography
LASSO: Least absolute shrinkage and selection operator
AUC: Area under curve
CA125: Carbohydrate antigen 125
DCA: Decision curve analysis
CA199: Carbohydrate antigen 199
AFP: Alpa-fetoprotein
CEA: Carcinoembryonic antigen
VOI: Volume of interest
ICC: Intra-group correlation coefficient
MPR: Multi-planar reformation
ROC: Receiver operating characteristic
GLSZM: Gray-level size zone matrix
GLDM: Gray-level dependence matrix
